# Direct and indirect role of non-coding RNAs in company with amyloid and tau protein in promoting neuroinflammation in post-ischemic brain neurodegeneration

**DOI:** 10.3389/fncel.2025.1670462

**Published:** 2025-10-31

**Authors:** Ryszard Pluta

**Affiliations:** Department of Pathophysiology, Medical University of Lublin, Lublin, Poland

**Keywords:** brain ischemia, neuroinflammation, non-coding RNAs, micro RNAs, circular RNAs, long non-coding RNAs, amyloid, tau protein

## Abstract

Post-ischemic brain neurodegeneration with subsequent neuroinflammation is a major cause of mortality, permanent disability, and the development of Alzheimer’s disease type dementia in the absence of appropriate treatment. The inflammatory response begins immediately after ischemia and can persist for many years. Post-ischemic neuroinflammation plays a dual role: initially, it is essential for brain repair and maintenance of homeostasis, but when it becomes uncontrolled, it causes secondary damage and worsens neurological outcome. Neuroinflammation is a complex phenomenon involving interactions between infiltrating immune cells from the peripheral circulation and resident immune cells in ischemic brain areas. This review focuses on the complex relationship between non-coding RNAs, amyloid accumulation, tau protein modifications, and the development of neuroinflammation in the post-ischemic brain. In particular, it clarifies whether the cooperation of non-coding RNAs with amyloid and tau protein enhances neuroinflammation and whether the *vicious cycle* of neuroinflammatory responses affects the production, behavior, and aggregation of these molecules. Ultimately, elucidating these interactions is critical, as they may contribute to resolving the phenomenon of post-ischemic brain neurodegenerative mechanisms. Furthermore, this review highlights the role of neuroinflammation as a functionally complex immune response regulated/mediated by transcription factors and cytokines. Additionally, it examines how the presence of non-coding RNAs, amyloid aggregation, and modified tau protein may shape the inflammatory landscape. This review aims to advance our understanding of post-ischemic neuroinflammation and its implications for long-term brain health.

## 1 Introduction

Human cerebral ischemia remains the leading cause of mortality and long-term disability worldwide (Pacheco-Barrios et al., 2022; Hou et al., 2024). The disease affects millions of people worldwide, placing a significant burden on those affected and on society (Ding Q. et al., 2022). In this regard, the United Nations has identified cerebral ischemia as a global priority to reduce the social burden (Ding Q. et al., 2022). The ultimate effect of cerebral ischemia is its neurodegeneration, a progressive phenomenon, classified as a neurodegenerative disease, meaning that it develops over time. Therefore, it is only several years after cerebral ischemia that patients notice symptoms such as memory loss and deterioration of cognitive functions. Survivors of ischemia often develop multiple cognitive impairments, ranging from mild cognitive impairment to advanced dementia (Dammavalam et al., 2024; Ngamdu and Kalra, 2024). It is worth emphasizing that the risk of dementia in people who have survived cerebral ischemia is twice as high as in people who have not had a history of ischemic injury (Ngamdu and Kalra, 2024). In addition, evidence suggests that post-ischemic brain damage accelerates the onset of dementia by about 10 years (De Ronchi et al., 2007; Pendlebury and Rothwell, 2009).

These symptoms occur because neurons in the hippocampus, responsible for thinking, learning and memory, die (Pluta et al., 2009; Pluta and Czuczwar, 2024a). As the disease progresses, there is a gradual loss of neuronal cells in other brain structures, ultimately leading to brain atrophy (Pluta et al., 2009). Amyloid plaques, neurofibrillary tangles, and cerebral amyloid angiopathy are characteristic features of progressive, post-ischemic brain neurodegeneration (Kato et al., 1988; Pluta et al., 1994a; Van Groen et al., 2005; Qi et al., 2007; Pluta et al., 2009; Hatsuta et al., 2019). Cerebral amyloid angiopathy is associated with cerebral vascular dysfunction, increased blood–brain barrier permeability, and chronic neuroinflammation, which further contributes to the progression of neurodegeneration with dementia of Alzheimer’s disease phenotype (Kiryk et al., 2011; Pluta et al., 2021a, 2023a; Pluta and Czuczwar, 2024b).

In recent years, a strong association has been demonstrated between the cumulative effects of cerebral ischemia and Alzheimer’s disease (Lecordier et al., 2022; Pluta, 2024). Cerebral vascular dysfunction leading to ischemic episodes is now widely recognized as an etiological factor of Alzheimer’s disease, causing the pathogenesis characteristic of Alzheimer’s disease (Elman-Shina and Efrati, 2022; Lecordier et al., 2022; Das et al., 2023; Pluta, 2024; Pluta, 2025). Neurofibrillary tangles together with amyloid plaques damage the functioning of neurons and their connections, which leads to the interruption of signal transmission in the neuronal network (Bloom, 2014). In addition, amyloid oligomers and plaques and neurofibrillary tangles activate neighboring microglial cells and astrocytes, which additionally leads to the multidirectional initiation of a neuroinflammatory reaction (Sobue et al., 2023; Pluta, 2025). The neuroinflammatory response, through the release of inflammatory factors, induces extensive, progressive acute and then chronic neuroinflammatory changes that additionally damage neighboring ischemic neurons, leading to their late death and severe damage to the neuronal network (Martínez-Cué and Rueda, 2020). Over time, the accumulation of these toxic substances and phenomena contributes to the progressive degeneration of neurons, which ultimately leads to a deterioration of cognitive functions and the onset of Alzheimer’s disease type dementia.

Post-ischemic neuroinflammation, which lasts from several minutes to several years, causes secondary irreversible damage to brain tissue (Sekeljic et al., 2012; Radenovic et al., 2020; Arruri and Vemuganti, 2022; Morris-Blanco et al., 2022; Pluta et al., 2021a; Pluta, 2025). For this reason, neuroinflammation is considered a key target for therapeutic intervention to improve post-ischemia outcomes (Lambertsen et al., 2019; Vemuganti and Arumugam, 2021). Post-ischemic neuroinflammation, which is associated with innate and adaptive immune responses, activates various cell types, matrix components, extra- and intracellular receptors, and related signaling events in the brain during acute, subacute, and chronic stages (Simats and Liesz, 2022). The inflammatory response of blood vessels exposes the adhesion molecule P-selectin on the surface of endothelial cells and platelets, which attracts circulating leukocytes to the endothelium, and P-selectin on platelets binds to leukocytes, forming intravascular emboli that secondarily impede blood flow and exacerbate cerebrovascular damage (Pluta et al., 1994b; Anrather and Iadecola, 2016). Activated endothelial cells also release E-selectin, vascular cell adhesion molecule 1 (VCAM-1), and intercellular adhesion molecule 1 (ICAM-1), which further support leukocyte binding and migration after cerebral ischemia (Andjelkovic et al., 2019).

On the other hand, the immune response in brain tissue is initiated by the release of damage-associated molecular patterns (DAMPs) such as high-mobility group box 1 (HMGB1), adenosine triphosphate (ATP), heat shock proteins (HSP), DNA, and RNA from dying cells, which are then detected by immune effectors, including brain-resident microglia, via pattern recognition receptors (Gülke et al., 2018). This recognition causes microglia to release inflammatory factors, including tumor necrosis factor alpha (TNF-*α*), interleukins (IL) IL-6, and IL-1β, inducible nitric oxide synthase (iNOS), complement proteins, and matrix metalloproteases (MMPs), leading to disruption of the integrity of the blood–brain barrier (Pluta et al., 2023a; Pluta, 2025). This allows peripheral immune cells, such as macrophages, neutrophils, T cells, and lymphocytes, to migrate to the ischemic-damaged area of the brain (Simats and Liesz, 2022). DAMPs associated with ischemic injury also induce reactive astrogliosis, which leads to the further release of inflammatory factors such as TNF-*α*, IL-1α, and interferon-*γ*, which enhance neuroinflammation and cause delayed neuronal death (Li L. et al., 2022). Although the acute neuroinflammatory response following cerebral ischemia aims to restore brain homeostasis, but uncontrolled chronic inflammation results in rapid neuronal cell death, leading to adverse neurological sequelae. The above phenomena indicate a dual role of neuroinflammation in ischemic injury (Jin et al., 2017; Pluta, 2025).

It should be emphasized that the transcription of pro- and anti-inflammatory genes in the ischemic brain is also tightly regulated by transcription factors and non-coding RNAs (ncRNAs) (Morris-Blanco et al., 2022; Pluta, 2025). For example, the transcription factor CCAAT/enhancer binding protein (C/EBP) *β*, considered one of the master regulators of the immune system, is activated in a subset of neurons within hours after cerebral ischemia (Pluta, 2025). This leads to the release of pro-inflammatory cytokines such as IL-1β and TNF-α (Wu et al., 2023). Another example is the induction of the proinflammatory transcription factor early growth response-1 (Egr1) after focal ischemia, which is known to induce inflammatory gene expression and secondary brain injury (Tureyen et al., 2008). Once inflammatory transcripts and cytokines are formed, their fate is controlled by interaction with ncRNAs and epitranscriptomic modifications, indicating the involvement of multiple levels of inflammatory gene regulation in the post-ischemic brain (Chokkalla et al., 2022).

Transcription factors not only control the induction of inflammatory factors, but also participate in the inflammatory cascade by regulating ncRNA expression. Among others, various transcription factors, including c-Myc, p53, NF-κB, and HIF-1α, are responsible for miRNA biogenesis and subsequent changes (Mehta et al., 2024a,b; Pluta, 2025). For example, the transcription factor p53 regulates miRNA-34a and miRNA-145 and thus influences the behavior of microglial cells (Mehta et al., 2024a,b). Similarly, NF-κB activation modulates the expression of many pro-inflammatory genes and miRNAs, such as miRNA-9, miRNA-21, miRNA-146a, and miRNA-155, which are known to influence inflammatory mRNAs (Adly Sadik et al., 2021; Zhan et al., 2023). In addition, transcription factor E2F1 can directly modulate miRNA-122 transcription in the ischemic brain. Moreover, changes in lncRNAs and circRNAs after cerebral ischemia are regulated by a set of transcription factors (Cao et al., 2020; Mehta et al., 2023a). Thus, NF-κB has recently been shown to exacerbate brain tissue damage following ischemia by promoting the expression of the pro-inflammatory lncRNA FosDT (Mehta et al., 2023a).

As research on the mechanisms of post-ischemia neurodegeneration progresses, increasing attention has been paid to the important role that previously overlooked ncRNAs may play. NcRNAs were initially thought to be “junk RNAs.” However, findings from the Human Genome Project and the ENCODE initiative have revealed that a significant portion of the human genome is transcribed into various ncRNAs (Watson, 1990; Djebali et al., 2012; ENCODE Project Consortium, 2012). These studies have shown that various ncRNAs play important roles in controlling gene expression and cellular mechanisms under both normal and pathological conditions. NcRNAs are a group of functional RNAs that do not encode proteins but regulate gene expression. The DNA sequence from which ncRNA is transcribed is often called an RNA gene. NcRNAs vary in size, shape, and location and are classified into three main types such as micro RNAs (miRNAs), long non-coding RNAs (lncRNAs), and circular RNAs (circRNAs) (Asim et al., 2021).

In the post-ischemic brain, ncRNAs have been shown to be involved in key processes related to ischemic pathology, including amyloid production, tau protein hyperphosphorylation, and neuroinflammation (Mehta et al., 2024a,b; Pluta, 2025). These unbalanced pathological processes cause secondary damage to the brain parenchyma after ischemia (Iadecola et al., 2020). An increasing number of studies indicate that ncRNAs influence the severity of pathological reactions by preventing the repair of brain parenchyma and at the same time causing progressive irreversible damage. Therefore, ncRNAs are currently considered as potential targets for future treatments of post-ischemic neurodegeneration (Vemuganti, 2013).

Moreover, the expression and distribution of ncRNAs in blood and brain are altered after ischemia, indicating the possibility of finding new biomarkers and potential prognostic factors after cerebral ischemia (Takuma et al., 2017). They interact with DNA, mRNA and proteins, which allows them to modulate many biological processes. New research has revealed a significant role of ncRNAs in the development of post-ischemic brain neurodegeneration, leading to irreversible neurological deficits.

This article reviews the role of ncRNAs in ischemia and reperfusion-induced brain injury, focusing on how ncRNAs influence key molecular and cellular pathways involved in the brain response to ischemia, with a primary focus on the development of neuroinflammation. The aim of this review is to synthesize the current research and provide a comprehensive understanding of the multifaceted role of ncRNAs after cerebral ischemia, which may provide a basis for future studies and new therapeutic strategies targeting ncRNAs. Although dysregulation of ncRNAs has been detected in the brain after ischemia, the mechanisms of how different types of ncRNAs regulate specific ischemia-related processes are still not fully understood.

It should be emphasized that this research gap limits our ability to fully understand the neuropathogenesis of neurodegeneration following cerebral ischemia. Therefore, the analysis of current studies on the relationship between ncRNAs and ischemia will determine the current state of knowledge and systematize the available knowledge on the mechanisms of progressive brain neurodegeneration after ischemia in connection with the development of neuroinflammation. Understanding the regulatory mechanisms of ncRNAs may not only shed light on the neurodegenerative processes following cerebral ischemia but also provide innovative biomarkers or therapeutic targets for the development of new diagnostic approaches and therapeutic strategies. These findings are expected to significantly accelerate the development of diagnostics and treatments for ischemia-induced brain neurodegeneration, improving patient outcomes and quality of life.

An additional aim of this review is to investigate the relationship between post-ischemic brain neurodegeneration and different types of ncRNAs in the context of amyloid accumulation and tau protein modification, with particular emphasis on the development of neuroinflammation, as well as to discover their functions and mechanisms in the negative interaction. Combining these phenomena may provide valuable information that will support future research in this area. The role of ncRNAs in the pathogenesis and search for targets for the prevention and treatment of post-ischemic brain neurodegeneration from the perspective of their properties has recently become a hot topic. To my knowledge, this is the first review paper linking three types of ncRNAs (circRNA, lncRNA, miRNA) in association with amyloid and tau protein in the development of neuroinflammation in post-ischemic brain neurodegeneration.

## 2 Non-coding RNAs post-ischemia

Numerous studies have revealed differential expression of miRNAs, lncRNAs, and circRNAs following cerebral ischemia and have revealed the processes by which specific ncRNAs modulate post-ischemia neurodegeneration (Dharap et al., 2012; Tiedt and Dichgans 2018; Zhang et al., 2019; Lu et al., 2020; Mehta et al., 2023b,c; Sun et al., 2026). Moreover, increasing evidence indicates an important role of ncRNAs in regulating the development of neuroinflammation after brain ischemia (Lu et al., 2020; Mehta et al., 2024a,b). The involvement of ncRNAs related to the development of neuroinflammation in the occurrence and progression of neurodegenerative processes following cerebral ischemia is increasingly being suggested. In recent years, with the development of knowledge about the role of ncRNAs after cerebral ischemia, they have begun to be treated as active rather than passive factors of neurodegeneration.

### 2.1 MiRNAs and post-ischemic neuroinflammation

It was revealed that some miRNAs, which are highly expressed in the ischemic brain, can be detected in blood. After regional cerebral ischemia, 114 miRNAs were identified. Only 10 miRNAs were differentially expressed in blood and brain tissue post-ischemia. MiRNAs expression pattern was shown to change upon reperfusion, indicating temporal manifestation during ischemic injury (Jeyaseelan et al., 2008). Growing evidence links miRNAs to secondary brain injury following cerebral ischemia (Dharap et al., 2009; Li G. et al., 2018; Pluta and Jabłoński, 2021). MiRNAs have been shown to regulate early and delayed stages of neuroinflammatory signaling following cerebral ischemia (Khoshnam et al., 2017).

Innate immune cells, including neutrophils, monocytes, macrophages, and NK cells, have been found to begin infiltrating the brain within hours of ischemia and release proinflammatory factors that promote neuroinflammation (Iadecola et al., 2020; Pluta, 2025). MiRNA-193a-5p has been shown to protect the brain after ischemia by restoring N2-type neutrophils by reprogramming them towards an anti-inflammatory phenotype (Han et al., 2023). In patients after focal cerebral ischemia, increased miR-193a-5p levels correlated with better neurological outcomes after 3 months of follow-up (Han et al., 2023). Similarly, miR-193a agomiR reduced infarct size and improved motor function after transient focal cerebral ischemia in mice, indicating a protective effect of miR-193a-5p (Han et al., 2023).

In patients after ischemia, reduced miR-424 concentration in blood was found, which correlated positively with neurological outcomes (Zhao et al., 2013). Mice subjected to permanent focal cerebral ischemia also showed reduced levels of miR-424 in the blood and ipsilateral cerebral hemisphere, and treatment with a miR-424 mimic inhibited microglia activation, resulting in neuroprotection (Zhao et al., 2013). In mice, after permanent or transient local cerebral ischemia, the level of miR-669c increased in the peri-infarct brain cortex (Kolosowska et al., 2020). Lentiviral overexpression of miR-669c decreased brain damage and improved neurological outcomes in mice after regional cerebral ischemia (Kolosowska et al., 2020). It was found that the positive effect of miR-669c was associated with the induction of alternative activation of microglia and macrophages (Kolosowska et al., 2020).

It was revealed that after reversible focal cerebral ischemia in mice, the level of miR-210 in the brain increased and was maintained for 7 days, and its inhibition suppresses TNF-*α*, IL-1 and IL-6 as well as reduced the infarct volume and improved motor functions (Lou et al., 2012; Huang et al., 2018). Abnormally regulated miRNAs have been found in blood even months after ischemic stroke in young patients. It has been shown that miRNAs can be used to differentiate stroke types. It has also been found that miRNAs profiling can provide an additional tool for clinicians to determine stroke outcome (Tan et al., 2009). It was found that miRNA-15a/miRNA-16-1 knockout mice showed resistance to reversible local cerebral ischemia (Yang et al., 2017). Additionally, antagomiRNA-15a/miRNA16-1 was shown to reduce brain damage and improve motor function after local cerebral ischemia (Yang et al., 2017). The obtained neuroprotective effects were associated with a reduction in the levels of TNF-*α*, IL-6, MCP-1 and VCAM-1 (Yang et al., 2017). Induction of miRNA-181 after focal ischemia promoted secondary brain injury, while its inhibition via suppression of NF-κB activity reduced infarct volume and improved behavioral recovery (Xu et al., 2015). Thus, several miRNAs have been shown to have the potential to regulate post-ischemic neuroinflammation and its associated secondary brain injury (Table 1).

**Table 1:** Regulatory role of non-coding RNAs in neuroinflammation in the post-ischemic brain.

NcRNA type	Target	Regulatory path	Effect	Brain ischemia	Reference
miRNA-181 ↑	NF-κB	Pro-inflammatory	Harmful	Focal	Xu et al. (2015)
miRNA-193a-5p ↑	N2-type neutrophils	Anti-inflammatory	Beneficial	Focal	Han et al. (2023)
miRNA-424 ↑	Inhibition microglia	Anti-inflammatory	Beneficial	Permanent focal	Zhao et al. (2013)
miRNA-669c ↑	Inhibition microglia & macrophages	Anti-inflammatory	Beneficial	Focal	Kolosowska et al. (2020)
miRNA-210 ↑	TNFα, IL-1β, IL-6 ↑	Pro-inflammatory	Harmful	Focal	Huang et al. (2018)
lncRNA H19 ↑	HDAC1-M1 microglia polarization	Pro-inflammatory	Harmful	Focal	Wang et al. (2017)
lncRNA MEG3 ↑	KLF4-M1 microglia polarization	Pro-inflammatory	Harmful	Focal	Li T. et al. (2020)
circRNA CDR1as ↓	TNFα, IL-1β ↑, α-synuclein pathology ↑	Pro-inflammatory	Harmful	Focal	Mehta et al. (2023b)
circRNA HCTD1 ↑	TNFα, IL-1β, IL-6 ↑	Pro-inflammatory	Harmful	Focal	Peng et al. (2019)

HDAC1, histone deacetylase 1; KLF4, Krüppel-like factor 4; NF-κB, nuclear factor kappa-light-chain-enhancer of activated B cells; TNFα, tumor necrosis factor α; IL-1β, Interleukin-1 beta; IL-6, Interleukin 6. Expression: ↑-increase, ↓-decrease.

### 2.2 LncRNAs and post-ischemic neuroinflammation

Clinical observations have revealed that lncRNAs are significantly altered in the blood and brains of patients with ischemic stroke and play a key role in its pathogenesis (Chen J. et al., 2021). Studies on the role and mechanisms of lncRNAs in the pathogenesis and recovery of stroke aim to promote the clinical application of lncRNAs as potential diagnostic and prognostic markers and therapeutic targets.

LncRNA FosDT induced after reversible focal cerebral ischemia has been shown to promote injury and behavioral deficits (Mehta et al., 2015). In contrast, silencing or knockdown the expression of the lncRNA FosDT gene after ischemia reduced the infarct size and improved motor functions (Mehta et al., 2015; Mehta et al., 2021a). The influence of lncRNA FosDT has been shown to be associated with the activation of inflammatory genes following ischemia (Mehta et al., 2015; Mehta et al., 2021a).

It has been shown that patients after cerebral ischemia have increased levels of lncRNA H19 in the blood, which promotes the development of neuroinflammation by driving histone deacetylase 1-dependent M1 microglial polarization (Wang et al., 2017). Moreover, after reversible focal cerebral ischemia in mice, increased levels of lncRNA H19 were demonstrated in white blood cells and brain (Wang et al., 2017). However, downregulation of lncRNA H19 after ischemia had a beneficial effect, reducing infarct size, cerebral edema, and improving motor function (Wang et al., 2017). It was shown that the above effect was caused by a decrease in the levels of TNF-*α* and IL-1β and an increase in the level of IL-10, which indicates the immunomodulatory role of lncRNA H19 post-ischemia (Wang et al., 2017). The above mechanism was confirmed in an *in vitro* ischemic model where it was shown that lncRNA H19 reduction changed the phenotype of microglial cells from pro-inflammatory to anti-inflammatory via inhibition of histone deacetylase 1 (Wang et al., 2017). Moreover, after reversible experimental local cerebral ischemia, lncRNA H19 was shown to promote leukocyte activation via the miRNA-29b/C1QTNF6 axis, triggering the release of TNF-α and IL-1β, which enhanced neuroinflammation (Li G. et al., 2022).

Microglia polarization has been shown to be influenced by lncRNA NEAT1, which is increased in the blood of patients after cerebral ischemia. Knockdown of lncRNA NEAT1 downregulates microglia activation, reduces neuronal apoptosis and infarct size after cerebral ischemia (Ni et al., 2020). Another study revealed reduced expression of lncRNA MALAT1 in the blood of patients after cerebral ischemia, which was closely associated with the negative effects of ischemia (Ren et al., 2020). However, high expression of lncRNA MALAT1 resulted in a reduction of the effects of cerebral ischemia and a decrease in the level of pro-inflammatory factors (Ren et al., 2020). Experimental studies after reversible cerebral ischemia revealed that lncRNA MALAT1 is upregulated in cerebral microvessels (Zhang et al., 2017). Mice with the lncRNA MALAT1 gene knocked out showed increased brain damage, neurological deficits and impaired motor function (Zhang et al., 2017). Increased levels of pro-inflammatory factors such as IL-6, E-selectin and MCP-1 were found in ischemic mice with the lncRNA MALAT1 gene knocked out (Zhang et al., 2017). The above studies provided evidence that lncRNA MALAT1 plays an important role after ischemia in reducing cerebrovascular and brain parenchyma damage through anti-apoptotic and anti-inflammatory activity.

In a subsequent study, lncRNA MEG3 was shown to be upregulated following ischemia and its downregulation promoted better neurological outcomes following reversible cerebral ischemia in animals (Yan et al., 2016; Li T. et al., 2020). The beneficial effect of lncRNA MEG3 silencing was associated with a change in microglial cell polarization towards an anti-inflammatory phenotype, as well as with a reduction in TNF-*α* and IL-1β levels, and a reduction in neuronal cell loss (Yan et al., 2016; Li T. et al., 2020). These studies indicate an important role of lncRNAs in the regulation of post-ischemic neuroinflammation and suggest their potential utility as therapeutic targets in the development of post-ischemic brain neurodegeneration (Table 1).

### 2.3 CircRNAs and post-ischemic neuroinflammation

CircRNAs have been shown to undergo quantitative and temporal changes in the peri-infarct brain cortex of animals following reversible ischemic brain injury (Mehta et al., 2017). It has been revealed that m6A-modified circRNAs can regulate functions related to synaptic processes, and this may potentially influence synaptic activity after cerebral ischemia (Mehta et al., 2024b). Among others, it has been presented that the level of circRNA CDR1as is downregulated during long-term reperfusion after transient focal ischemia (Mehta et al., 2017; Mehta et al., 2023b). However, increased expression of circRNA CDR1as was found to provide resistance to cerebral ischemic injury by increasing the level of miRNA-7 and inhibiting the pathological effects of α-synuclein (Mehta et al., 2023c). The data therefore indicate that miRNA-7 is a pro-survival molecule essential for the recovery of brain function following ischemia (Mehta et al., 2023b,c). The neuroprotective effect of circRNA CDR1as seems to be partially mediated by reducing the level of IL-1β released from activated microglial cells, which has a direct effect on limiting the severity of neuroinflammation after ischemia (Mehta et al., 2023b). There is a study indicating that overexpression of circRNA CDR1as promotes the transition of macrophages to an anti-inflammatory phenotype, indicating the anti-inflammatory properties of circRNA CDR1as (Gonzalez et al., 2022).

In a study of blood mononuclear cells from patients with cerebral ischemia, increased expression of circRNA HECTD1 in these cells was demonstrated, which correlated with blood levels of TNF-α, IL-6, and IL-1β. Expression of circRNA HECTD1 correlated with higher disease risk, disease severity, neuroinflammation, and recurrent cerebral ischemia (Peng et al., 2019). Moreover, it was revealed that the concentrations of circRNA FUNDC1, circRNA PDS5B and circRNA CDC14A in blood were significantly increased in patients after cerebral ischemia, and their levels correlated with the infarct size (Zuo et al., 2020). In patients after cerebral ischemia, a significant increase in the expression of circRNA PDS5B in lymphocytes and granulocytes and only in granulocytes for circRNA CDC14A was found, and their levels positively correlated with the infarct volume. Taken together these three circRNAs enhance neuroinflammatory responses following brain ischemia. The combination of expression of these 3 circRNAs may serve as a biomarker for diagnosing and predicting stroke outcomes (Zuo et al., 2020). High expression of circRNA CDC14A was revealed in blood and peri-infarct neutrophils and astrocytes in mice after reversible local cerebral ischemia. Decreased expression of circCDC14A in peripheral blood neutrophils reduced significantly surface area of activated astrocytes in the peri-infarct cortex, infarct size and neurological outcome and survival rate significantly improved within 7 days (Zuo et al., 2021). Overall, the above evidence indicate an important role of circRNAs in regulating post-ischemic neuroinflammation (Table 1).

## 3 CircRNA-miRNA interactions in post-ischemic neuroinflammation

CircRNAs bind and control miRNAs, and this interaction controls competitive endogenous RNA (ceRNA) networks (circRNA–miRNA–mRNA) to regulate translation of target mRNAs. CircRNA ciRS-7/CDR1as, which is highly expressed in neocortical and hippocampal neurons, is an example of a circRNA with a regulatory role in the ceRNA network (Hansen et al., 2011). Other studies indicate that the circRNA/mRNA network plays a key role in controlling neuroinflammation in neurodegeneration (Piwecka et al., 2017; Mehta et al., 2020; Mehta et al., 2023b). CircRNA polymorphisms associated with neuroinflammation development have been shown to be associated with functional outcomes in patients after cerebral ischemia (Liu et al., 2021). For example, the circ-STAT3 rs2293152 GG genotype was identified as associated with poorer recovery 90 days after ischemia. The results indicate that circ-STAT3 may be a novel biomarker for predicting functional outcomes after ischemic stroke and an important contributor to post-ischemic recovery (Liu et al., 2021).

It was found that the levels of circRNA DLGAP4 in blood were significantly reduced in both patients and mice after focal cerebral ischemia (Bai et al., 2018). Upregulation of circRNA DLGAP4 significantly attenuated neurological deficits and reduced infarct volume and blood–brain barrier permeability in transient focal cerebral ischemia in mice. Data indicate that circRNA DLGAP4 ameliorates the effects of cerebral ischemia by targeting miR-143 (Bai et al., 2018). Another study revealed that the expression of circRNA TTC3 was upregulated in mice after focal cerebral ischemia (Yang et al., 2021). Depletion of circRNA TTC3 reduced the infarct volume, neurological deficits, and brain water content. In this study, it was found that circRNA TTC3 promoted post-ischemic brain injury via the miR-372-3p/TLR4 axis (Yang et al., 2021). One day after ischemia, circRNA_0000831 was shown to be significantly reduced in the mouse brain (Huang et al., 2022). In contrast, overexpression of the circRNA_0000831 inhibited neuroinflammation and apoptosis by blocking miRNA-16-5p (Huang et al., 2022).

CircRNA CDC14A was shown to be induced in *in vivo* and *in vitro* ischemia models (Huo et al., 2022). In contrast, silencing circRNA CDC14A reduced cerebral infarction, apoptosis, and neurological deficits induced by local cerebral ischemia. Thus, circCDC14A promoted post-ischemic brain injury via regulating the miR-23a-3p/CXCL12 axis, suggesting that circCDC14A may become a potential therapeutic target for cerebral ischemia (Huo et al., 2022).

Transient focal cerebral ischemia in mice significantly reduced the levels of both circRNA CDR1as and miR-7 in the peri-infarct cortex within 3–72 h of recirculation (Mehta et al., 2023b). Overexpression of circRNA CDR1as inhibited *α*-synuclein generation after ischemia, accelerated motor function recovery, reduced infarct size, and reduced markers of apoptosis, autophagy, mitochondrial fragmentation, and neuroinflammation. Stimulation of circRNA CDR1as was shown to exert neuroprotective effects after ischemia, likely by protecting miRNA-7 and preventing α-synuclein-induced neuronal death (Mehta et al., 2023b). Transient cerebral ischemia has been shown to increase α-synuclein gene expression and protein levels in neurons (Kim T. et al., 2016; Pluta et al., 2025a). It has also been shown that knocking down or knocking out *α*-synuclein significantly reduced neuroinflammation, infarct volume, and promoted neurological recovery in rodents subjected to cerebral ischemia (Kim T. et al., 2016). Moreover, increased miRNA-7a-5p level suppressed α-synuclein translation, leading to improved neuronal survival after experimental focal cerebral ischemia (Kim et al., 2018; Mehta et al., 2023c). It has been noted that the relationship between circRNA CDR1as and miRNA-7 is quite complex, namely, instead of inhibiting its function, circRNA CDR1as can protect, stabilize and transport miRNA-7 in cells, suggesting that interactions between circRNA CDR1as and miRNAs are important for proper brain function (Piwecka et al., 2017; Mehta et al., 2023b). Data indicate that interactions between circRNAs and miRNAs play a key role in the modulation of neuroinflammation in post-ischemic brain neurodegeneration.

## 4 Interaction of different ncRNAs in post-ischemic neuroinflammation

Various types of ncRNAs, including lncRNAs, circRNAs and miRNAs, form regulatory networks that influence gene expression and cellular processes, thus playing an important role in regulating physiological and pathological processes in the brain (Mehta et al., 2021b). Many lncRNAs have been found to be dysregulated in patients after cerebral ischemia, and in experimental models of cerebral ischemia, they have been shown to act as competing RNAs that remove mRNAs and thus control many post-ischemic neuropathological processes, including neuroinflammation (Bhattarai et al., 2017; He et al., 2018; Zhang et al., 2020). Expression of lncRNA SNHG14 was increased in mice with focal cerebral ischemia. Silencing of lncRNA SNHG14 reduced ischemic brain damage by inhibiting neuroinflammation and also brain edema via miR-199b/miRNA-145/AQP4 axis (Qi et al., 2017; Wang et al., 2020; Zhang G. et al., 2021; Zhang Z. et al., 2021; Zhang H. et al., 2021).

In patients after brain ischemia, synchronized overexpression of lncRNA H19 and tumor necrosis factor C1q-related protein 6 (C1QTNF6) and decreased expression of miRNA-29b were found in leukocytes (Li G. et al., 2022). Similar changes in the expression of lncRNA H19 and miRNA-29b were found after the first day of recirculation following local brain ischemia in rats (Li G. et al., 2022). It has been shown that miRNA-29b can bind C1QTNF6 mRNA and suppress its expression, while lncRNA H19 can prevent the suppression of C1QTNF6 expression by acting as a sponge for miRNA-29b, thereby maintaining the expression of C1QTNF6. Overexpression of C1QTNF6 promoted the release of TNF-*α* and IL-1β in leukocytes, further increased the permeability of the blood–brain barrier and aggravated ischemic brain injury (Li G. et al., 2022). These results confirm that lncRNA H19 promotes leukocyte activity by targeting the miRNA-29b/C1QTNF6 axis in post-ischemic brain injury (Li G. et al., 2022). In another study, lncRNA Tug1 was shown to contribute to NLRP3 inflammasome-dependent pyroptosis after cerebral ischemia via the miRNA-145a-5p/Tlr4 axis (Yao et al., 2022). However, inhibition of lncRNA Tug1 after experimental cerebral ischemia led to reduced microglial cell activity, reduced infarct size, and improved neurological outcomes (Yao et al., 2022).

In patients after cerebral ischemia, the expression of lncRNA UCA1 increased and miRNA-18a-5p decreased (Yan et al., 2023). Knockdown of lncRNA UCA1 showed a protective role by reducing infarct size, neurological deficits, and neuroinflammation in rats via increased expression of miRNA-18a-5. MiRNA-18a-5p was involved in the regulation of lncRNA UCA1 on cell viability, cell apoptosis, and neuroinflammation. In patients after cerebral ischemia, overexpression of lncRNA UCA1 and under expression of miR-18a-5p showed an inverse correlation (Yan et al., 2023).

Moreover, inhibition of lncRNA ANRIL after ischemia using siRNA showed beneficial effects on neuroinflammation after focal cerebral ischemia in mice by increasing the expression of miRNA-671-5p (Deng et al., 2022). Inhibition of lncRNA ANRIL reduced the levels of TNF-*α*, IL-1β, IL-6, and NF-κB and preserved the expression of tight junction proteins, affecting the restriction of blood–brain barrier permeability in mice after focal cerebral ischemia (Deng et al., 2022). Downregulation of lncRNA ANRIL attenuates neuroinflammation in the middle cerebral artery occlusion-reperfusion model by modulating the miR-671-5p/NF-κB pathway.

Studies of patients after cerebral ischemia have shown the influence of reduced expression of lncRNA ZFAS1 in mononuclear cells on the development of neuroinflammation, neurological impairment and survival (Wang et al., 2022). In contrast, high expression of lncRNA ZFAS1 was associated with low levels of TNF-α, IL-1β and IL-6. The explanation may be that lncRNA ZFAS1 reduces the expression of miRNA-582 to decrease the production of pro-inflammatory cytokines, thereby attenuating inflammation in patients post-ischemia (Wang et al., 2022). In contrast, it has been shown that exosomes released from bone marrow stem cells which carry the lncRNA ZFAS1, can limit oxidative stress and neuroinflammation associated with ischemia by inhibiting miRNA-15a-5p (Yang and Chen, 2022). There are data indicating that genetic deletion of endothelial miRNA-15a/16-1 attenuated blood–brain barrier pathology, reduced infarct size and decreased infiltration of peripheral immune cells after ischemia (Ma et al., 2020). These mice also showed reduced infiltration of pro-inflammatory M1-type microglia/macrophages in the peri-infarct area, without changes in the number of proliferating M2-type cells. Elucidation of the molecular mechanisms of miRNA-15a/16-1-dependent blood–brain barrier dysfunction may enable the discovery of novel therapies for cerebral ischemia (Ma et al., 2020). Since endothelial claudin-5 plays a key role in blood–brain barrier permeability after ischemia, inhibition of miRNA-15a directly or indirectly via overexpression of lncRNA ZFAS1 may be a novel strategy to limit the infiltration of the ischemic lesion by peripheral immune cells.

The expression of lncRNA HCG11 was found to be increased after local reversible cerebral ischemia (Gao et al., 2022). Studies with lncRNA HCG11 downregulation showed that it inhibited the growth of neuroinflammatory factors, limited infarct size, and improved neurological outcomes after ischemia (Gao et al., 2022). LncRNA HCG11 was shown to affect and negatively regulate miRNA-381-3p in the post-ischemic brain (Gao et al., 2022). However, co-inhibition of miR-381-3p with antagomir and lncRNA HCG11 with siRNA exacerbated post-ischemic brain injury, which was reduced by siRNA alone. These effects of lncRNA HCG11 and miRNA-381-3p were mediated by p53, a key regulatory factor in Wnt signaling and necrosis/apoptosis (Gao J. et al., 2023). It has been shown that loss of p53 can cause the production of Wnt ligands, which stimulate macrophages to produce IL-1*β*, which leads to the development of neuroinflammation (Wellenstein et al., 2019; Gao C. et al., 2023). The presented studies show that silencing of lncRNA HCG11 protects the brain from neurodegenerative damage after ischemia by regulating p53 through miR-381-3p (Gao et al., 2022). Taken together, the results of the above studies clearly indicate that the interaction between lncRNA and miRNA modulates mRNA function and neuroinflammation after cerebral ischemia.

## 5 Cross-talk of circRNAs, miRNAs, and lncRNAs in post-ischemic neuroinflammation

The complexity of ceRNA networks increases when lncRNAs and circRNAs compete for a single miRNA target, indicating an additional dimension of genetic regulation (Kleaveland et al., 2018; Mehta et al., 2021b). Targeting of miRNAs by other ncRNAs can have opposing effects. For example, circRNA CDR1as and lncRNA Cyrano compete with each other for miRNA-7a-5p in the brain (Kleaveland et al., 2018). LncRNA Cyrano induces a structural change in miRNA-7a-5p recognized by ZSWIM8 Cullin-RING, leading to its proteolysis and exposure of miRNA-7a-5p to cytoplasmic nucleases (Shi et al., 2020). Degradation of miRNA-7a-5p after ischemia leads to increased *α*-synuclein levels and neuroinflammation (Kim et al., 2018; Mehta et al., 2023c). Thus, investigating the interplay of different classes of ncRNAs in controlling neuronal phenomena and thus pathological events such as neuroinflammation leads to the identification of potential mechanisms for designing new therapeutic targets to improve post-ischemic outcomes (Table 1).

## 6 MiRNAs influence amyloid production and clearance

Amyloid production and aggregation are essential for the progression of post-ischemic brain neurodegeneration. Some studies have revealed the role of miRNAs in regulating amyloid production, metabolism and elimination (Sun et al., 2026). In recent years, the following miRNAs: miRNA-106, miRNA-520c, miRNA-101, miRNA-20a, miRNA-17-5p, miRNA-106b, miRNA-16, miRNA-135a, miRNA-200b, miRNA-153, miRNA-298, miRNA-342-5p and miRNA-346 have been shown to have the ability to regulate the level of amyloid precursor protein (Patel et al., 2008; Hébert et al., 2009; Vilardo et al., 2010; Long et al., 2012; Massone et al., 2012; Liu et al., 2014; Long et al., 2019; Chopra et al., 2021; Dong et al., 2022; Sun et al., 2026).

MiRNAs play a key role in regulating the activity of the three main secretases involved in the metabolism of amyloid precursor protein and in amyloid production, i.e., α-secretase, β-secretase and *γ*-secretase (Table 2) (Sun et al., 2026). Under normal conditions, α-secretase triggers the non-amyloidogenic pathway by cleaving the amyloid precursor protein at the alpha site, thereby preventing amyloid formation (Lichtenthaler and Haass, 2004). MiRNA-30a-5p inhibits the production of non-amyloidogenic proteins by inhibiting α-secretase, thereby contributing to the increased level of amyloid peptide 1–42 (Sun et al., 2022). In addition, miRNA-144 reduces the level of α-secretase by blocking the transcriptional and translational processes of α-secretase (Sun et al., 2017).

**Table 2:** Regulatory role of non-coding RNAs in amyloid production from amyloid precursor protein.

NcRNA type	Target	Regulation of amyloid	Effect	Reference
miRNA-30-a-5p ↑	α-secretase ↓	Amyloid ↑	Harmful	Sun et al. (2022)
miRNA-144 ↑	α-secretase ↓	Amyloid ↑	Harmful	Sun et al. (2017)
miRNA-29a ↓	β-secretase ↑	Amyloid ↑	Harmful	Hébert et al. (2008)
miRNA-29b-1 ↑	β-secretase ↓	Amyloid ↓	Beneficial	Hébert et al. (2008)
miRNA-186 ↓	β-secretase ↑	Amyloid ↑	Harmful	Kim J. et al. (2016)
miRNA-298 ↑	APP ↓, β-secretase ↓	Amyloid ↓	Beneficial	Chopra et al. (2021)
miRNA-107 ↓	β-secretase ↑	Amyloid ↑	Harmful	Wang et al. (2008)
miRNA-34a-5p, miRNA-125b-5p, ↑	β-secretase ↓	Amyloid ↓	Beneficial	Li P. et al. (2020)
miRNA-340 ↑	β-secretase ↓	Amyloid ↓	Beneficial	Tan et al. (2020)
miRNA-34a ↓	γ-secretase ↓	Amyloid ↓	Beneficial	Jian et al. (2017)
miRNA-3940-5p ↑	Presenilin 1 ↓	Amyloid ↓	Beneficial	Qi et al. (2024)
lncRNA-BC200 ↓	β-secretase ↓	Amyloid ↓	Beneficial	Li H. et al. (2018)
lncRNA-BACE1-AS ↑	β-secretase ↑	Amyloid ↑	Harmful	Zeng et al. (2019)
circRNA Cwe27 ↑	Pur-α ↓, APP ↑	Amyloid ↑	Harmful	Song et al. (2022)
circRNA-AXL ↑	β-secretase ↑	Amyloid ↑	Harmful	Li L. et al. (2022)
circRNA RS-7 ↓	Ubiquitin-26S proteasome clearance system ↓	Amyloid accumulation and senile plaque ↑	Harmful	Zhao et al. (2016)

Pur-α, Purine-rich element-binding protein A; APP, amyloid precursor protein. Expression: ↑-increase, ↓-decrease.

In contrast, beta-site amyloid precursor protein cleaving enzyme 1 (BACE1) acts as a β-secretase, cleaving the N-terminal region of the amyloid precursor protein, resulting in the formation of the amyloid-containing C99 fragment. The C99 fragment is then cleaved by γ-secretase (Takasugi et al., 2023). Increased expression of miRNA-29a and miRNA-29b-1 has been shown to lead to decreased expression of BACE1, resulting in reduced amyloid production (Hébert et al., 2008). MiRNA-186 and miRNA-298, reduce levels of human amyloid precursor protein and BACE1 which result in lower amyloid levels (Kim J. et al., 2016; Chopra et al., 2021). However, reduced expression of the miRNA-107 positively affects the level of BACE1, which results in increased amyloid levels (Wang et al., 2008). Upregulation of miRNA-34a-5p, miRNA-125b-5p, and miRNA-340 leads to downregulation of BACE1 expression and reduced amyloid accumulation (Li T. et al., 2020; Tan et al., 2020).

*Γ*-secretase, which consists of at least presenilin 1 and 2, also plays a key role in the processing of amyloid precursor protein into amyloid (Hur, 2022). Studies with a lack of miRNA-34a have shown improved cognitive performance in transgenic mice by inhibiting γ-secretase activity but without affecting β- and α-secretase activity (Jian et al., 2017). Another study showed the inhibition of presenilin 1, a key component of γ-secretase by miRNA-3940-5p (Qi et al., 2024). Furthermore, inhibition of miR-4536-3p has been shown to reduce amyloid accumulation and tau protein phosphorylation in the brain (Choi et al., 2025).

It has been shown that the elimination of harmful amyloid from the brain in neurodegenerative diseases, also after ischemia, occurs via autophagy (Ułamek-Kozioł et al., 2016; Ułamek-Kozioł et al., 2017; Ułamek-Kozioł et al., 2019; Limanaqi et al., 2020; Pluta et al., 2024; Tang et al., 2024; Pluta et al., 2025b). In the brains of Alzheimer’s disease patients, miRNA-7 deficiencies were found to be associated with a deficiency of the ubiquitin-conjugating enzyme UBE2A, which is necessary for amyloid degradation (Zhao et al., 2016). Following ischemia, the lysosomal pathway involved in autophagy has been shown to play a critical role in recycling cellular components by degrading excess or damaged organelles and misfolded proteins, including amyloid. This process maintains cellular homeostasis by converting misfolded proteins, such as amyloid, into their basic components, thereby preserving cellular integrity (Zhang Z. et al., 2021). During the progression of neurodegeneration, including after ischemia, changes in the miRNA regulation of autophagy-related proteins are crucial for the occurrence and development of pathology. In a transgenic model of Alzheimer’s disease, the expression of miRNA-331-3p and miRNA-9-5p was found to be decreased in the early stage of the disease and increased in the late stage (Sun et al., 2026). MiRNA-331-3p and miRNA-9-5p have been shown to interact with the autophagy receptors sequestosome 1 and optineurin, respectively (Chen M. L. et al., 2021). It was observed that overexpression of miRNA-331-3p and miRNA-9-5p in SH-SY5Y cell line impaired autophagy activity and promoted the accumulation of amyloid plaques. In contrast, in a mouse model of Alzheimer’s disease, enhanced amyloid clearance, improved cognitive functions and mobility were demonstrated after treatment with miR-331-3p and miR-9-5p antagonists in the late stage of the disease (Chen M. L. et al., 2021). Research indicates that the use of miRNA-331-3p and miRNA-9-5p, along with autophagy activity and amyloid plaques, may allow for the differentiation of early and late stages of Alzheimer’s disease, which will translate into a more accurate and faster diagnosis.

In contrast, silencing the miRNA-140 gene repressed the development of Alzheimer’s disease in a rat model. This was associated with increased autophagy, which prevented mitochondrial dysfunction by silenced miRNA-140 (Liang et al., 2021). Intracerebroventricular administration of agomiRNA-299-5p in Alzheimer’s disease mouse model inhibited both autophagy and apoptosis and improved cognitive performance of mice. Results suggest that miRNA-299-5p modulates neuronal survival programs by regulating autophagy (Zhang et al., 2016). However, miRNA-101a has been shown to have a negative effect on the regulation of autophagy (Li et al., 2019). It has been observed that inhibition of autophagy activity by overexpression of miRNA-23b may be associated with the improvement of cognitive functions after brain injury (Sun et al., 2018).

## 7 MiRNAs influence tau protein hyperphosphorylation and clearance

In post-ischemic neurodegeneration, tau protein undergoes changes mainly through phosphorylation, which adversely affects the condition of neurons and contributes to the formation of neurofibrillary tangles (Kato et al., 1988; Hatsuta et al., 2019; Khan et al., 2018; Pluta et al., 2021b, 2022a,b; Pluta and Czuczwar, 2024b). MiRNAs have been shown to influence tau protein levels by modulating MAPT gene expression (Table 3) (Piscopo et al., 2023; Sun et al., 2026). It has been found that the deficiency of miRNA-132/212 and miRNA-219 disrupts the metabolism of tau protein and promotes its pathological aggregation in neurons, which ultimately leads to the development of neurofibrillary tangles (Santa-Maria et al., 2015; Smith et al., 2015).

**Table 3:** Regulatory role of non-coding RNAs in tau protein modification.

NcRNA type	Target	Regulation of tau protein	Effect	Reference
miRNA-219 ↓	GSK-3β phosphorylation ↑	Expression and aggregation ↑	Harmful	Santa-Maria et al. (2015)
miRNA-132/212 ↓	GSK-3β, PP2B phosphorylation ↑	Expression, aggregation and insoluble form ↑	Harmful	Smith et al. (2015)
miRNA-23b-3p ↑	GSK-3β phosphorylation ↓	Amyloid tau protein interaction ↓	Beneficial	Sun et al. (2026)
miRNA-128 ↑	GSK-3β, APPBP2, mTOR ↓	Amyloid deposition and tau protein interaction ↓	Beneficial	Li et al. (2023)
miRNA-539-5p ↑	GSK-3β phosphorylation ↓	Amyloid deposition and tau protein interaction ↓	Beneficial	Jiang et al. (2020)
lncRNA NEAT1 ↑	FZD3/GSK3β/pathway phosphorylation ↓	Microtubule stabilization by tau protein	Beneficial	Zhao et al. (2020)
lncRNA MALAT1 ↑	CDK5 phosphorylation ↑	Aggregation and neurofibrillary tangles ↑	Harmful	Spreafico et al. (2018)
lncRNA-00507 ↑	Hyperphosphorylation by P25/P35/GSK3β ↑	Aggregation ↑	Harmful	Yan et al. (2020)
lncRNA ZBTB20-AS1 ↑	GSK-3β phosphorylation ↑	Aggregation ↑	Harmful	Wang Y. et al. (2023)
circRNA AXL ↑	Phosphorylation ↑	Aggregation ↑	Harmful	Li Y. et al. (2020)

GSK-3β, glycogen synthase kinase-3β; PP2B, protein phosphatase 2B; APPBP2, amyloid beta precursor protein binding protein 2; CDK5, cyclin-dependent kinase 5. Expression: ↑-increase, ↓-decrease.

Glycogen synthase kinase-3β has been shown to play a multifaceted role in cerebral ischemia–reperfusion injury (Li et al., 2024). MiRNA-23b-3p has been shown to protect against amyloid-induced tau protein hyperphosphorylation by acting on GSK-3β, thereby protecting neurons from programmed death (Sun et al., 2026). In cells treated with the miRNA-23b-3p analogue, a significant decrease in tau protein phosphorylation at serine-396 and serine-404 residues was observed, as well as a decrease in the production of amyloid peptide 1–42 (Sun et al., 2026). MiRNA-539-5p has been shown to bind directly to GSK-3β, leading to reduced GSK-3β activity and consequently reduced amyloid accumulation in the brain (Sun et al., 2026). Also miRNA-128 inhibits tau protein phosphorylation by inhibiting GSK3β expression and as a result reduces amyloid accumulation (Li et al., 2023).

Cyclin-dependent kinase 5 (CDK5) has been shown to cause increased phosphorylation of tau protein, leading to increased tau protein accumulation and toxicity, which ultimately leads to the development of neurofibrillary tangles (Saito et al., 2019). It was presented that melatonin alleviates tau protein-related pathologies via upregulation of miRNA-504-3p and p39/CDK5 axis (Chen et al., 2022). In other studies, miRNA-103/107, miRNA-124 and miRNA-26a were found to inhibit CDK5 expression in neurodegeneration and control apoptosis (Farina et al., 2017; Angelopoulou et al., 2019; Sun et al., 2026).

Elevated microtubule affinity-regulating kinases (MARKs) differentially regulate tau protein missorting and amyloid-dependent synaptic pathology and are closely linked to early tau protein phosphorylation in the brains of Alzheimer’s disease patients (Lund et al., 2014; Chudobová and Zempel, 2023). MiRNA-515-5p and miRNA-582-3p were revealed to act as suppressors of microtubule affinity regulating kinase gene expression and inhibited apoptosis via regulation of the miRNA-582-3p/MARK3 axis (Pardo et al., 2016; Wang L. et al., 2023). Moreover, the degradation of phosphorylated tau protein is largely regulated by the autophagy pathway, in which miRNA-9 plays an important role by reducing its phosphorylation (Subramanian et al., 2021).

## 8 LncRNAs influence amyloid production and clearance

It has been shown that patients with Alzheimer’s disease have elevated levels of lncRNA SORL1 in the brain, which influences the formation of amyloid and is a risk factor for the development of this disease (Ciarlo et al., 2013; Mishra et al., 2023). Silencing of lncRNA BC200 was shown to suppress BACE1 expression, and overexpression of lncRNA BC200 increased BACE1 expression and enhanced the formation of amyloid peptide 1–42 (Table 2). Furthermore, inhibition of lncRNA BC200 increased cell viability and reduced cell apoptosis in an Alzheimer’s disease model by directly targeting BACE1 (Li H. et al., 2018). In a subsequent study, lncRNA BACE1-AS was shown to prevent BACE1 mRNA degradation by sequestering BACE1-targeting miRNAs (Zeng et al., 2019). Another study showed that increasing the level of lncRNA BACE1-AS led to the inhibition of miR-485-5p, which in turn reduced the inhibition of BACE1 mRNA (Faghihi et al., 2010). In contrast, the study of lncRNA BDNF-AS revealed that through the regulation of the miR-9-5p/BACE1 pathway, it enhanced neurotoxicity in Alzheimer’s disease by promoting the formation of amyloid plaques (Ding Y. et al., 2022). This indicates a key role of lncRNA BDNF-AS in the occurrence and development of Alzheimer’s disease. Subsequently, lncRNA NEAT1 was shown to act as a promoting factor for Alzheimer’s disease progression via modulation of the miRNA-124/BACE1 axis (Zhao et al., 2019). Furthermore, silencing of lncRNA XIST reduced the Alzheimer’s disease-associated alteration of BACE1 via miR-124 (Yue et al., 2020). Recent facts imply that interaction between lncRNA CYP3A43-2/miRNA-29b-2-5p and PSEN1 affected its activity and decreased amyloid plaque development and improved cognitive function (Wuli et al., 2022).

The low-density lipoprotein receptor protein 1 (LRP1) in neuroglial cells forms complexes with amyloid and removes it into the extracellular space (Shinohara et al., 2017; Faissner, 2023; Pluta et al., 2023b). Increased expression of lncRNA LRP1-AS and decreased expression of LRP1 were found in patients with Alzheimer’s disease. Increased levels of lncRNA LRP1-AS were shown to be associated with decreased stability and inhibited translation of LRP1 mRNA, which impairs amyloid clearance by LRP1 (Yamanaka et al., 2015). Another study revealed that lncRNA BACE1-AS promotes neuronal damage by autophagy via miRNA-214-3p/ATG5 signaling axis (Zhou et al., 2021). In contrast, suppression of miR-214-3p reversed the effects of lncRNA BACE1-AS and ATG5 on amyloid peptide 1–42-induced cellular damage (Sun et al., 2026).

## 9 LncRNAs influence tau protein phosphorylation

Studies in neurodegeneration, lncRNA NEAT1, lncRNA HOTAIR, and lncRNA MALAT1, revealed their effects on CDK5R1 expression, and this affected CDK5 activity, which is associated with tau protein phosphorylation (Table 3) (Spreafico et al., 2018). LncRNA NEAT1 and lncRNA HOTAIR were shown to negatively regulate CDK5R1 mRNA levels, while lncRNA MALAT1 had a positive effect. It was also found that all three lncRNAs positively controlled the miRNA-15/107 family. LncRNA NEAT1 had increased expression levels in the temporal cortex and hippocampus of Alzheimer’s disease patients. It is suggested that the upregulated lncRNA NEAT1 in Alzheimer’s disease brain probably acts as part of a protective mechanism against neuronal death (Spreafico et al., 2018). Moreover, lncRNA NEAT1 was shown to regulate microtubule stabilization via the FZD3/GSK3*β*/P-tau pathway in APP/PS1 mice (Zhao et al., 2020). The above findings revealed that lncRNA NEAT1 affects tau protein hyperphosphorylation in neurodegenerative processes (Zhao et al., 2020). Another study showed that lncRNA 00507 also regulates tau protein hyperphosphorylation in neurodegenerative diseases via the miRNA-181c-5p/TTBK1/MAPT axis (Yan et al., 2020). Subsequent study showed that overexpression of lncRNA ZBTB20-AS1 inhibited ZBTB20 expression and increased GSK-3β expression and tau protein phosphorylation (Table 3) with apoptosis, which promoted the progression of Alzheimer’s disease (Wang Y. et al., 2023). Data indicate that long non-coding RNAs play a key role in the pathogenesis of neurodegenerative diseases by controlling amyloid and tau protein (Yang et al., 2025).

## 10 CircRNAs influence amyloid production and tau protein status

The circRNA HDAC9/miRNA-138/sirtuin-1 pathway mediates amyloidogenic processing of amyloid precursor protein, amyloid accumulation and its neurotoxicity resulting in synaptic dysfunction and the development of cognitive deficits in Alzheimer’s disease (Lu et al., 2019). Furthermore, it was revealed that circRNA Cwc27 was upregulated in neurons and brains of APP/PS1 mice, as well as in temporal cortex and blood of Alzheimer’s disease patients (Song et al., 2022). Silencing circRNA Cwc27 reduced neuropathological changes in Alzheimer’s disease and alleviated cognitive deficits. Moreover, a novel RNA-binding protein-dependent regulatory axis was identified, where circRNA Cwc27 interacted with purine-rich element-binding protein *α* and trapped it in the cytoplasm, resulting in its inactivation and transcriptional upregulation of amyloid precursor protein and other Alzheimer’s disease-related genes (Song et al., 2022). Knockdown of circRNA_0004381 reduced hippocampal neuronal injury and promoted microglia M2-type polarization by the miR-647/PSEN1 axis, ultimately improving cognitive function in a mouse model of Alzheimer’s disease (Li N. et al., 2022). Deficiency of the circRNA RS-7, which downregulates the expression of the ubiquitin-conjugating enzyme UBE2A and affects amyloid clearance by proteolysis, has been shown to be depleted in the brains of Alzheimer’s disease patients, leading to amyloid accumulation and the formation of amyloid plaques (Zhao et al., 2016). It was further shown that increased expression of circRNA hsa_circ_0131235 in the temporal cortex was closely associated with the neuropathology of Alzheimer’s disease (Bigarré et al., 2021). In addition, in patients with Alzheimer’s disease, circRNA-AXL and circRNA-GPHN correlated negatively, while circRNA-PCCA and circRNA-HAUS4 correlated positively with Mini-mental State Examination scores (Li Y. et al., 2020). CircRNA-AXL correlated negatively, while circRNA-PCCA, circRNA-HAUS4, and circRNA-KIF18B correlated positively with β-amyloid peptide 1–42 levels. CircRNA-AXL and circRNA-GPHN correlated positively, while circRNA-HAUS4 correlated negatively with total tau protein. CircRNA-AXL correlated positively with phosphorylated tau protein (Table 3) (Li Y. et al., 2020).

Exosome treatment improved cognitive function by delivering circRNA-Epc1 and changing the M1/M2 polarization of microglial cells in a mouse model of Alzheimer’s disease. As a result, neuroinflammatory factors and neuronal apoptosis in the hippocampus were reduced (Liu et al., 2022). In contrast, circRNA NF1-419 increased autophagy in astrocytes via PI3K-I/Akt-AMPK-mTOR and PI3K-I/Akt–mTOR signaling pathways and affected inflammatory mediators and delayed the development of dementia in an animal model of Alzheimer’s disease (Diling et al., 2019). It was revealed that circRNA circ_0000950 in Alzheimer’s disease promoted neuronal apoptosis and increased the expression of inflammatory cytokines via miRNA-103 sponge (Yang et al., 2019). But circRNA AXL increased neuronal damage and neuroinflammation via miRNA-328’s effect on BACE1 in Alzheimer’s disease (Li et al., 2022a). It was shown that circRNA LPAR1 promoted Aβ25-35-induced apoptosis, neuroinflammation, and oxidative stress by the miRNA-212-3p/ZNF217 axis (Wu et al., 2021).

Nrf2 was revealed to enhance hippocampal synaptic plasticity, learning, and memory by the circRNA-Vps41/miRNA-26a-5p/CaMKIV regulatory network (Zhang et al., 2022). Overexpression of circRNA-Vps41 positively affected synaptic plasticity and memory dysfunction via the miRNA-24-3p/Synaptophysin axis (Li et al., 2022b). These findings revealed the regulatory network of circRNA-Vps41 and provided new insights into its potential to improve learning and memory in association with aging (Li et al., 2022b). It should be noted that in patients with Alzheimer’s disease, a sex-dependent deregulation of circRNA HOMER1 variants was revealed in the entorhinal cortex (Urdánoz-Casado et al., 2021). N6-methyladenosine-modified circRNA RIMS2 was shown to mediate synaptic and memory impairments in Alzheimer’s disease by activation of UBE2K-dependent GluN2B ubiquitination and degradation via sponging miRNA-3968 (Wang L. et al., 2023). Recently, circular RNAs and exosomes have been shown to play an important role in amyloid and tau protein pathologies in Alzheimer’s disease (Pala and Yilmaz, 2025).

## 11 Amyloid versus neuroinflammation

Studies have shown a marked activation of neuroglial cells that exhibit an inflammatory phenotype in post-ischemic brain neurodegeneration, especially in the vicinity of amyloid deposits (Pluta et al., 2009; Swardfager et al., 2014). Ultrastructural studies have revealed that microglia accumulate and direct their processes towards amyloid deposits, indicating a direct interaction between microglia and amyloid and suggesting that amyloid accumulation is the main driving force for microglial cell activation (Wisniewski et al., 1992). It has been found that microglia can take up amyloid via their processes and store it in endosomes. This event increases the size and number of microglia in proportion to the surface area of amyloid deposits (Ebrahimi et al., 2025). Amyloid can bind to microglial surface receptors, such as the receptor for advanced glycation end products (RAGE), thereby inducing an inflammatory signaling pathway (Ebrahimi et al., 2025). In addition, positron emission tomography confirmed this link, showing an increased number of activated microglial cells in the brains of people with Alzheimer’s disease, and this increase was directly correlated with cognitive decline (Okello et al., 2009). Activated microglia release pro-inflammatory cytokines such as tumor necrosis factor alpha, interleukin 6, and 1β, as well as cytotoxic molecules such as nitric oxide and reactive oxygen species, leading to neuronal cell damage (AmeliMojarad and AmeliMojarad, 2024). In the post-ischemic brain, similarly to Alzheimer’s disease, chronic activation of glial cells near amyloid deposits is consistently observed (Pluta et al., 1994a; Pluta et al., 2009; Swardfager et al., 2014; Ding et al., 2025), and the release of pro-inflammatory factors from these activated cells exacerbates disease progression (Gao J. et al., 2023; AmeliMojarad and AmeliMojarad, 2024; Pluta, 2025). On the one hand, microglia can phagocytize and degrade amyloid. On the other hand, prolonged exposure to amyloid triggers the release of pro-inflammatory factors that cause chronic neuroinflammation, contributing to synaptic dysfunction, myelin damage, neuronal cell death, and cognitive impairment (Swardfager et al., 2014; Zhang H. et al., 2021; Gao J. et al., 2023; AmeliMojarad and AmeliMojarad, 2024; Chiarini et al., 2025; Hong et al., 2025; Patel et al., 2025).

Activated microglia have an unrestricted influence on the scope and severity of neuroinflammation and the spread of abnormal protein aggregates like amyloid (Chiarini et al., 2025; Yuasa-Kawada et al., 2026). Increased levels of apoptosis-associated speck-like protein containing a caspase recruitment domain are observed following microglial activation (Yuasa-Kawada et al., 2026). ASC is an important adaptor protein involved in the inflammasome, playing a critical role in the innate immune response to inflammatory stimuli. ASC also forms speckles through self-oligomerization in response to pathology, a key step in inflammasome activation and pyroptosis (a form of programmed cell death). Accumulated ASC molecules are released from glial cells and taken up by neighboring cells, causing the inflammation to spread. Extracellular ASC specks bind to amyloid, providing a core for amyloid seeding/cross-seeding, thereby amplifying amyloid pathology like neuroinflammation (Yuasa-Kawada et al., 2026). Amyloid-ASC composite fibrils have been shown to increase amyloid toxicity in microglial cells, leading to their death (Friker et al., 2020).

The presence of inflammatory mediators is clearly increased in the vicinity of amyloid deposits and neurofibrillary tangles, but on the other hand, these factors are known to promote the production of amyloid peptides (Chiarini et al., 2025; Ebrahimi et al., 2025). Neuroinflammatory processes are further enhanced by key pro-inflammatory factors that promote amyloid accumulation, thus exerting a cytotoxic effect on neuronal cells (Swardfager et al., 2014; Patel et al., 2025). Chronic activation of glial cells, especially microglia and astrocytes, leads to the continuous production of proinflammatory factors (Sekeljic et al., 2012; Radenovic et al., 2020), driving neurodegenerative processes and causing cognitive dysfunction, as demonstrated in animal models of cerebral ischemia and observed in patients after brain ischemia (Kiryk et al., 2011; Cohan et al., 2015; Rost et al., 2022; Pluta and Czuczwar, 2024a). Major pathological phenomena such as microgliosis, astrogliosis and amyloid accumulation have been shown to be inextricably linked to cognitive decline and progression of neurodegeneration after ischemia (Pluta et al., 1997; Pluta et al., 2009; Sekeljic et al., 2012; Swardfager et al., 2014; Radenovic et al., 2020).

**Figure 1:**
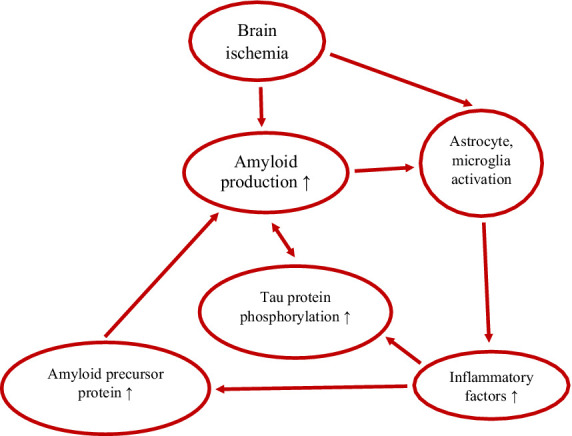
Vicious cycle between amyloid, tau protein and inflammation in post-ischemic brain neurodegeneration. ↑-increase.

Microglia interact with amyloid through both innate immune responses and antibody-dependent responses (Ebrahimi et al., 2025). Microglial cells phagocytize fibrillar amyloid via binding to innate immune receptors on their surface, whereas soluble amyloid is engulfed via LRP 1 and micropinocytosis (Ebrahimi et al., 2025). IL-1β in microglia and astrocytes has been shown to induce the production of amyloid precursor protein, leading to the formation of more amyloid deposits and plaques and increased neuroinflammation (Figure 1) (Chiarini et al., 2025; Ebrahimi et al., 2025). In addition, IL-1β promotes the secretion of amyloid-binding proteins such as α1-antichymotrypsin and apolipoprotein E by astrocytes, which triggers amyloid aggregation (Ebrahimi et al., 2025). Microglial cells and astrocytes have been found to accumulate around amyloid deposits and become highly activated upon attachment to amyloid plaques (Zhang G. et al., 2021; Zhang Z. et al., 2021; Zhang H. et al., 2021; Chiarini et al., 2025; Ebrahimi et al., 2025).

## 12 Tau protein versus neuroinflammation

The initiation and development of tau protein pathology in the brain after ischemia (Kato et al., 1988; Wen et al., 2004; Khan et al., 2018; Hatsuta et al., 2019) and subsequent neurodegeneration depend mainly on microglial cells and inflammatory factors (Swardfager et al., 2014; Chiarini et al., 2025; Patel et al., 2025). Abnormal function of microglial and astrocytic cells phagocytizing different neuronal compartments exacerbates tau protein pathology (Chiarini et al., 2025; Ebrahimi et al., 2025). In addition, microglia and astrocytes help spread tau protein, which leads to the progression of tau protein pathology and its spread to various brain structures (Chiarini et al., 2025; Ebrahimi et al., 2025). Tau protein aggregates localize in the vicinity of microglia and directly activate them (Ebrahimi et al., 2025; Hong et al., 2025). Tau protein aggregates change microglia morphology and induce their secretion of pro-inflammatory factors. Pathological tau protein activity begins concurrently with amyloid neurotoxicity (Chiarini et al., 2025; Ebrahimi et al., 2025). In the meantime, tau protein leads to increased amyloid accumulation (Figure 1) (Ebrahimi et al., 2025). Ultimately, amyloid-tau protein synergy, with the help of neuroinflammatory factors (Sekeljic et al., 2012; Radenovic et al., 2020), leads to synaptic dysfunction and neuronal death and post-ischemic neurodegeneration, identical to Alzheimer’s disease (Pluta et al., 2009; Swardfager et al., 2014; Guan et al., 2022; Pluta, 2024; Ebrahimi et al., 2025; Pluta, 2025). Post-ischemic brain neuroinflammation is generally believed to result from the amyloid cascade in response to neurofibrillary tangles (Kato et al., 1988; Hatsuta et al., 2019; Guan et al., 2022; Chiarini et al., 2025; Ebrahimi et al., 2025).

Interferon-induced transmembrane protein 3 has been shown to be significantly increased in astrocytes following cerebral ischemia and plays a key role in modulating the neuroinflammatory response and amyloids production (Ni et al., 2025). Increased production of amyloid peptides 1–40 and 1–42 results from increased *γ*-secretase activity induced by increased expression of interferon-induced transmembrane protein 3 in astrocytes (Ni et al., 2025).

Both astrocytic and microglial cells contribute to the neuroinflammatory response observed in post-ischemic brain neurodegeneration (Figure 1), similar to that seen in Alzheimer’s disease (Swardfager et al., 2014; Chiarini et al., 2025; Ding et al., 2025; Li L. et al., 2025; Patel et al., 2025; Pluta, 2025). Reactive astrocytes and microglia release inflammatory mediators that contribute to chronic neuroinflammation (Li L. et al., 2025; Pluta, 2025). In addition, astrocytes are involved in the removal of amyloid, while microglia play a key role in the phagocytosis of amyloid plaques what influences neuroinflammation (Li L. et al., 2025; Pluta, 2025). In post-ischemic neurodegeneration, ongoing neuroinflammation causes amyloid accumulation (Figure 1) (Chiarini et al., 2025), which affects astrocytic and microglial cells dysfunction and deepens communication problems between neurons, which in turn impairs cognitive function (Kiryk et al., 2011; Cohan et al., 2015; Pluta and Czuczwar, 2024a). Moreover, amyloid-activated ASC specks increase the expression of tau protein kinases in microglia and influence the aggregation of hyperphosphorylated tau protein in neuronal cells (Ising et al., 2019; Chiarini et al., 2025). The above effect is enhanced by tau protein monomers/oligomers released from neurons. Phagocytosis by microglial cells, secretion, and uptake of exosomes causes the spread of tau protein between neurons. Therefore, phagocytosis of microglial cells and their aggregative (cross-) spreading combined with neuroinflammation enhance neurodegeneration (Yuasa-Kawada et al., 2026). Analysis of neuropathological events in which neuroinflammation occurs in response to amyloid deposition has shown an association with hyperphosphorylated tau protein aggregation, which contributes to disease progression by further increasing neuroinflammation (Figure 1) (Yuasa-Kawada et al., 2026). Thus, non-coding RNAs play an important role in the pathogenesis of brain neurodegeneration by interacting with amyloid, tau protein, and neuroinflammation (Jiménez-Ramírez and Castaño, 2025).

## 13 Conclusions and perspectives

Acute cerebral ischemia in humans is a clinical emergency and a condition associated with significant morbidity, mortality and disability. Accurate prognostic and predictive biomarkers and therapeutic targets for acute cerebral ischemia and post-ischemic neurodegeneration remain undefined. This review explores the multifaceted interactions between ncRNAs, amyloid and tau protein and their contribution to the pathological picture of post-ischemic brain neurodegeneration, including neuroinflammation as a secondary insult. The accumulation of amyloid and tau protein, as well as the neuroinflammatory reaction in brain cells, triggers neurodegenerative processes, which thus accelerate the progression of the disease. Thus, recent advances in ncRNAs and genetics research have allowed for more detailed insight into the interactions between amyloid, tau protein, and the immune response.

It is important to note that this pioneering review highlights the limitations in the availability of studies and findings due to their lack of data and suggests a path forward for the presented research. Currently, no study provides data on survival over 2 years after cerebral ischemia, although other studies suggest the feasibility of such studies (Radenovic et al., 2020; Pluta, 2025). Such a long survival time would allow for the definition of a therapeutic window during post-ischemic brain neurodegeneration. Previous genetic studies (e.g., on APP, tau protein, autophagy, mitophagy, apoptosis) indicate that a dramatic increase in genetic changes occurs after approximately one year of ischemia (Czuczwar et al., 2024; Pluta, 2024; Pluta et al., 2024; Pluta et al., 2025b). If we had ncRNAs data from this survival period, they could indicate a therapeutic window for post-ischemia brain neurodegeneration, i.e., the presumed time for effective treatment. This would facilitate the search for the appropriate timing of action, but such data are currently lacking. In the absence of a defined therapeutic window, the most effective timing of drug administration is currently a matter of speculation. Studies with very long animal survival times, i.e., up to 2 years, may allow for long-term assessment of genetic changes and increase their accuracy and validity, given that current cerebral ischemia studies terminate their observations after several days. Therefore, the data originate from the beginning of the acute phase of changes. Long-term studies would allow us to determine the pattern of changes in gene expression related to ncRNAs and amyloid and tau protein, given that symptoms of post-ischemic brain neurodegeneration, including dementia, appear after approximately 10 to 20 years of survival. Furthermore, it is well known that 2 years of age in rodents corresponds to approximately 80 years in humans. Data on ncRNAs in different brain structures at different survival times are lacking. These observations require verification in studies lasting up to 2 years in different models of cerebral ischemia in different animals, which would allow for the correlation of genetic, proteomic, and neuropathological changes. This would enable a precise assessment of the progression of the complexity of dysregulation at the genomic and proteomic levels from the acute to the chronic phase of ischemic brain neurodegeneration.

Understanding the differential roles of ncRNAs, amyloid, tau protein, and transcription factors in the development of neuroinflammation in the post-ischemic brain is crucial for the development of targeted therapies. Studying the interactions between neurodegeneration, immune response and cell death opens promising perspectives for the development of innovative treatment methods. However, despite decades of research, the basic mechanisms underlying post-ischemic neurodegeneration are still not fully understood, although new ones are emerging all the time. A deeper understanding of the mechanisms of neurodegeneration following ischemia and the identification of promising biomarkers and therapeutic targets will be crucial to advancing treatment and care. This review discusses post-ischemic neuroinflammation as a functionally complex immune cell response coordinated by several transcription factors and regulated at the molecular level by ncRNAs and intracellular epitranscriptome changes in association with the presence of amyloid and tau protein. This highlights the importance of further studies aimed at understanding the molecular mechanisms of post-ischemic pathophysiology and identifying new targets to develop treatment strategies aimed at minimizing ischemic brain damage and accelerating recovery. Therefore, therapies aimed at temporally regulating the neuroinflammatory cascade following ischemia may yield better outcomes.

Neuroinflammation is a major driver of post-ischemic brain neuropathology. Chronic post-ischemic neuroinflammation increases neuronal damage, promotes amyloid and plaque accumulation and tau protein dysfunction, and contributes to cognitive impairment and the development of dementia. Current clinical treatment for ischemic brain injury focuses on restoring cerebral blood flow and reoxygenation. These methods do not address the mechanisms underlying ischemic damage, including oxidative stress, apoptosis, blood–brain barrier permeability, and neuroinflammation. To reverse the changes and disability following cerebral ischemia, causal drugs are needed that affect the molecular and cellular processes induced by ischemia. Post-ischemic neuroinflammation, necessary to remove amyloid, tau protein, and cellular debris to prepare the brain for repair, plasticity, and regeneration, can be harmful if prolonged. Chronic neuroinflammation exacerbates secondary brain damage, impairs tissue repair, and contributes to progressive post-ischemic neurodegeneration and dementia.

Modulation of post-ischemia gene expression, e.g., amyloid precursor protein and tau protein, leads to neuroinflammation, delayed neuronal death, and adverse outcomes. However, the functioning of these phenomena in post-ischemic brain neurodegeneration also depends on their interaction with ncRNAs controlling the expression of genes responsible for post-ischemic neuroinflammation. Various ncRNAs, including miRNAs, lncRNAs, and circRNAs, have been shown to be essential regulators of gene expression in ischemia and post-ischemic neuroinflammation. Moreover, RNA modifications following ischemia (epitranscriptomics) influence gene expression and protein synthesis, also affecting neuroinflammation. RNA modification modulates the biogenesis and splicing of ncRNAs, influencing neuroinflammation, e.g., by polarizing the neuroglial cell state. Thus, manipulation of epitranscriptomic marks may prove to be a potential means of regulating the neuroinflammatory response, accelerating tissue repair, and improving functional recovery after ischemia. In short, the interplay of transcription factors, ncRNAs, and epitranscriptomic changes is responsible for post-ischemia neuroinflammation, thereby regulating secondary ischemia-induced brain injury and functional outcomes. A deeper understanding of these mechanisms is crucial for identifying new targets and developing therapeutic strategies aimed at ameliorating post-ischemic neurological dysfunction and facilitating functional recovery.

**Figure 2:**
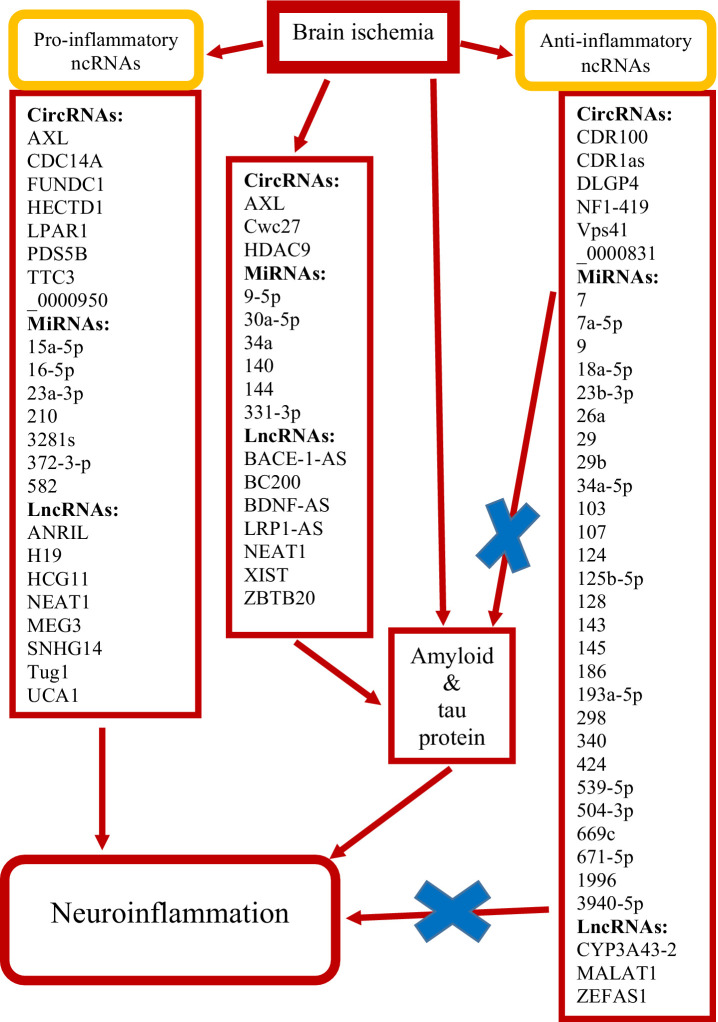
Summary of non-coding RNAs (ncRNAs) identified as potentially protective (×) and exacerbating brain neuropathology following ischemia. Circular RNAs (circRNAs), long non-coding RNAs (lncRNAs) and micro RNAs (miRNAs).

Following cerebral ischemia, endothelial cells release molecules that promote the adhesion and migration of peripheral immune cells across the blood–brain barrier into the ischemic area. The immune response in the ischemic area is initiated by neuroglial cells. In addition, amyloid and tau protein are involved in the above processes, which enhance neuroinflammation after ischemia (Figure 2). This is a novel insight, pointing to an interplay between amyloid, tau protein, and neuroinflammation in promoting post-ischemia neurodegeneration. Ultimately, this cascade of events activates transcription factors that regulate gene expression, cytokine production, neuronal survival and death, and neurological outcome.

This review presents the involvement of three types of ncRNAs: miRNAs, lncRNAs and circRNAs in the development of neuroinflammation and the pathogenesis of post-ischemic neurodegeneration in close cooperation with amyloid and tau protein. This article details how these three ncRNAs participate in pathological events in the post-ischemic brain, such as amyloid production, tau protein phosphorylation, and neuroinflammatory responses in a vicious cycle. Furthermore, the potential use of ncRNAs as diagnostic markers and therapeutic targets is analyzed. These studies indicate an important role of ncRNAs in understanding the relationship with the development of post-ischemic brain neurodegeneration. This study describes the potential pathogenesis of post-ischemic neurodegeneration in relation to ncRNAs and their products and key regulatory mechanisms. It also highlights the current challenges in clinical diagnostics and treatment of ncRNAs, such as issues with delivery mechanisms, precise targeting, and ensuring treatment efficacy (Li S. et al., 2025). Nevertheless, our knowledge about the regulatory functions of ncRNAs in the context of post-ischemic neurodegeneration is still limited and many aspects require further investigation.

It should be noted that the activity of these factors in the post-ischemic brain is modulated by their mutual interactions, accessibility to DNA binding sites, and cross-talk influences of ncRNAs. NcRNAs, including miRNAs, lncRNAs, and circRNAs, have emerged as essential regulators of gene expression in post-ischemic neuroinflammation. With the advances in high-throughput gene sequencing analysis of clinical samples and experimental models, many aberrantly expressed ncRNAs have been detected in the brain and blood after acute ischemia (Dykstra-Aiello et al., 2016; Li S. et al., 2025). It has been shown that differentially expressed ncRNAs in the post-ischemic brain can lead to neuroprotection or deterioration (Figure 2), and therefore ncRNAs may serve as therapeutic targets for acute cerebral ischemia in humans and animals (Dykstra-Aiello et al., 2016; Li S. et al., 2025).

Moreover, ncRNAs, through their presence in blood, can be used as biomarkers to predict the development and sequelae of acute cerebral ischemia. Recently, the presence of ncRNAs in peripheral immune cells has been demonstrated to be involved in the systemic and cerebral immune response following acute brain ischemia (Li S. et al., 2025). This review explores the latest knowledge on ncRNAs (miRNAs, lncRNAs, and circRNAs) involved in the phenomena following cerebral ischemia, as well as the potential use of these ncRNAs as biomarkers in predicting and prognosticating the outcomes of acute ischemic episodes. The data from this review provided new ideas for the clinical application of ncRNAs after acute brain ischemia. Parallel studies on the mechanisms of ischemic brain neurodegeneration have focused on genes encoding proteins associated with Alzheimer’s disease (Kocki et al., 2015; Pluta et al., 2016a,b, 2018, 2020, 2024, 2025a–c; Czuczwar et al., 2024).

However, over the last decade, attention has also been paid to the key role of ncRNAs in neurodegenerative processes in the brain after ischemia (Mehta et al., 2024a; Li S. et al., 2025). NcRNAs are functional RNA molecules that are not translated into proteins. Generally speaking, ncRNAs can be divided into housekeeping ncRNAs and regulatory ncRNAs. Housekeeping ncRNAs are essential for basic cellular functions, while regulatory ncRNAs are crucial in gene expression which affects various cellular activities in different conditions. NcRNAs have been shown to play key roles in immunity, cell proliferation, apoptosis, oxidative stress, amyloid production and aggregation, tau protein phosphorylation, autophagy, and neuroinflammation, which have a profound impact on the development of post-ischemic brain neurodegeneration (Pluta, 2024; Li S. et al., 2025). They constitute a class of RNA molecules that, do not encode proteins, interact with DNA, RNA, proteins and even other ncRNAs to modulate a wide range of biological processes such as gene transcription, RNA turnover, mRNA translation and protein splicing.

MiRNA, lncRNA and circRNA have attracted the attention of scientists due to their interaction with various molecular phenomena and their crucial role in regulating gene expression (Dykstra-Aiello et al., 2016; Canoy et al., 2024; Mehta et al., 2024a; Li S. et al., 2025). In particular, miRNAs have been found to influence the expression of genes involved in amyloid production and deposition, tau protein phosphorylation, and neuroinflammation. LncRNAs have been observed to regulate amyloid production and tau protein phosphorylation. Interestingly, they have also been found to bind to miRNAs or proteins and can therefore modulate their activity. In the same sense, circRNAs have been observed to act as miRNA sponges. Hence, ncRNAs offer new tools for predicting and treating the post-ischemic brain, and their mechanisms of action and role in cerebral ischemia and post-ischemic brain neurodegeneration deserve further investigation. Research on the regulation of ncRNAs in post-ischemic neurodegeneration is now extensive, but several key issues still remain to be clarified. First, there have been few studies that have synthesized different types of ncRNAs and the networks they form.

In general, interactions between ncRNAs are extremely complex and diverse, and this review mainly focuses on the influence of individual ncRNAs on processes after cerebral ischemia. Studies conducted so far have rarely taken into account the network formed by different categories of ncRNAs and their collective impact on target processes. Therefore, more comprehensive studies are needed to elucidate the exact functions of these complex regulatory networks and feedback loops in post-ischemia neurodegeneration. Second, the role of ncRNAs at different stages of post-ischemic neurodegeneration remains unclear, especially with respect to changes in their expression and association with disease pathology from early to late clinical stages. It is crucial to investigate the temporal dynamics and stage-specific role of ncRNAs in the progression of neurodegeneration after ischemia. Moreover, although the mechanisms of action of many ncRNAs regulating post-ischemic neurodegeneration have been initially identified, much of this knowledge remains speculative and requires confirmation in *in vivo* and *in vitro* studies.

Several ncRNAs show potential as biomarkers of post-ischemic neurodegeneration, but their clinical significance requires confirmation in large-scale, long-term studies. Furthermore, it is necessary to develop standard protocols for the detection and quantification of ncRNAs. Finally, although ncRNA-based therapies have potential, they face challenges related to their efficacy, specificity, and safety of administration. Developing effective drug delivery systems and ensuring precise, controlled regulation in the brain remain fundamental hurdles. In summary, the relationship between post-ischemic neurodegeneration and ncRNAs is a promising area of research. NcRNAs offer new insights into the pathogenesis of post-ischemic neurodegeneration and the discovery of new biomarkers and therapeutic targets. Ongoing studies are expected to fully elucidate the role of ncRNAs regulatory networks in post-ischemic neurodegeneration, leading to more effective strategies for its prevention and treatment.

The effect on the epitranscriptome offers hope for regulating neuroinflammation, preventing amyloid accumulation and modifying tau protein, promoting tissue repair and improving neurological outcomes after brain ischemia. Influence of ncRNAs and epitranscriptomic mechanisms suggests potential new therapeutics in post-ischemic brain neurodegeneration. For example, targeting miRNA-7 and lncRNA FosDT showed improved results in experimental models, indicating a promising direction for future treatments for neurodegeneration (Mehta et al., 2024a; Pluta, 2025). Certainly, further experimental studies are needed to clarify the role of RNA modifications, RNA processing, RNA–RNA and RNA-protein interactions in post-ischemic neuroinflammation. Epitranscriptomic RNA modifications acting on ischemia affect functional outcomes. Precise manipulation of these processes will be crucial in developing future effective post-ischemia therapies.

There is much evidence that the treatment of neuroinflammation has a future and opens up tempting possibilities for medical intervention that patients after cerebral ischemia are waiting for. However, translating preclinical results from experimental studies into effective clinical therapies is currently a huge challenge (Candelario-Jalil and Paul, 2021). Present actions center on modifying the neuroinflammatory answer to move immune and neuroglial cells towards an anti-inflammatory phenotype, thereby helping the survival of ischemic neuronal cells (Mehta et al., 2024a; Pluta, 2025). This approach appears promising as a therapeutic method to ameliorate ischemia and recirculation-induced injury. Such actions will enhance our ability to mitigate neuroinflammation and also reduce the global burden of post-ischemic brain neurodegeneration. For example miRNA-155 is known to play a key role in promoting post-ischemic brain damage, including apoptosis, neuroinflammation, and microglia/astrocyte polarization, suggesting that miRNA-155 inhibitors may be attractive drugs for the treatment of acute phase of ischemia that can be combined with currently used standard treatments. Therefore, research based on pharmacomodulation and/or genetic manipulation of ncRNAs and epitranscriptomic regulators is actively conducted to develop new treatment methods. Some studies involving ncRNA manipulation, such as miRNA-7 and lncRNAs FosDT and lncRNA MALAT1, have been shown to improve outcomes after experimental cerebral ischemia (Kim et al., 2018; Wang et al., 2020; Mehta et al., 2023a,c). NcRNA-based drugs are in the early stages of development and face many challenges, including specificity, delivery, and tolerability, which require much further research. This review presents the neuroinflammatory response in association with ncRNAs in post-ischemic brain neurodegeneration. It is certain that reducing harmful neuroinflammation can help reduce the effects of cerebral ischemia. Moreover, to determine an effective therapeutic strategy, it is important to investigate the mechanisms between ncRNAs and other neuroinflammatory factors such as amyloid and modified tau protein.

This review highlights the crucial role of ncRNAs in the molecular processes of post-ischemic brain neurodegeneration, emphasizing their potential as biomarkers and therapeutic targets. As ncRNA research continues to expand, there is hope that new strategies for diagnosing, treating, and managing cerebral ischemia will be discovered, which may lead to better outcomes for people affected by this devastating neurodegenerative disease. The data in this review indicate that ncRNAs may be not only promising biomarkers for the early detection of cerebral ischemia but also novel therapeutic targets. More detailed studies on the role of ncRNAs in post-ischemic brain injury are certainly needed to determine the role of these potential biomarkers as predictive and therapeutic targets. It is important to determine the mechanism of action of ncRNAs and to determine how they can serve as treatment targets in the long term. As shown in this review, preliminary data on ncRNAs in the development of post-ischemic neurodegeneration have provided valuable information on the molecular mechanisms driving neurodegenerative processes. These observations point the way to the development of targeted interventions that can potentially modulate ncRNA activity, thereby opening new avenues for the treatment and management of post-ischemic brain neurodegeneration. Furthermore, this review clearly indicates the importance of advancing the knowledge of ncRNAs in cerebral ischemia through further studies. Thus, it is essential to confirm the role of ncRNAs in a wide range of experimental and clinical studies to strengthen their usefulness as clinical biomarkers and therapeutic agents in cerebral ischemia.

Ischemic brain injury is also associated with secondary neuronal cell death due to chronic neuroinflammation, induced by multiple mechanisms, both known and unknown. Research findings regarding these changes could help us better understand the neuropathogenesis and mechanisms of ischemic brain injury and bridge the gap between basic and translational research, potentially leading to the development of new therapeutic approaches to the treatment of neurodegeneration. Substances considered as drugs should be characterized by ease of administration, low off-target effects, and easy penetration of the blood–brain barrier. Moreover, elucidation of the complex network of interactions between different types of ncRNAs and their combined effect on the pathology of cerebral ischemia may reveal new, previously unknown mechanisms of this disease.
